# Supplementary material for: Causal Effects of Prenatal Exposure to PM2.5 on Child Development and the Role of Unobserved Confounding
Source: Int J Environ Res Public Health. 2019 Nov 9;16(22):4381. doi: 10.3390/ijerph16224381 (PMC6888495; doi:10.3390/ijerph16224381)
Supplement: Supplementary file 1 [file ijerph-16-04381-s001.pdf]

Table S1. Range of the estimated propensity score among treated and controls, by imputation.

	Number of subjects		Min, max propensity score	
	Controls	Treated	Controls	Treated
First imputation	178	213	(0.008, 0.971)	(0.042, 0.984)
Second imputation	193	198	(0.004, 0.980)	(0.030, 0.992)
Third imputation	190	201	(0.009, 0.969)	(0.033, 0.988)
Fourth imputation	183	208	(0.001, 0.958)	(0.014, 0.987)
Fifth imputation	189	202	(0.005, 0.978)	(0.038, 0.989)

Table S2. Between imputation variance, within imputation variance and their ratio, calculated for the average causal effect of treatment on the treated (ATT) for mental and psychomotor scores.

Mental score			Psychomotor score		
Variance			Variance		
Between imputation	Within imputation	B/W (%)	Between imputation	Within imputation	B/W (%)
0.43	8.04	5.4	0.83	9.12	9.1

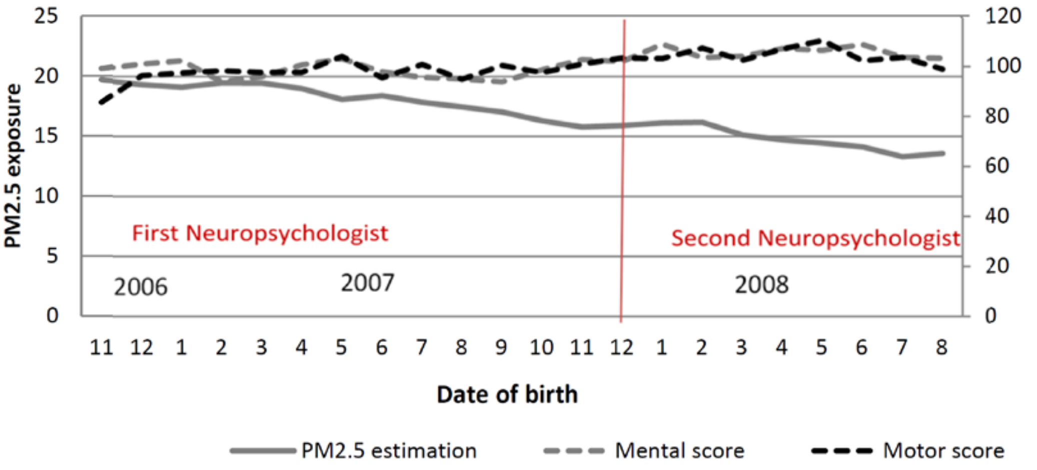

Figure S1. Time series of monthly average $PM_{2.5}$ exposures and monthly average mental and psychomotor scores. The red line indicates the time when the second neuropsychologist substituted the first one.

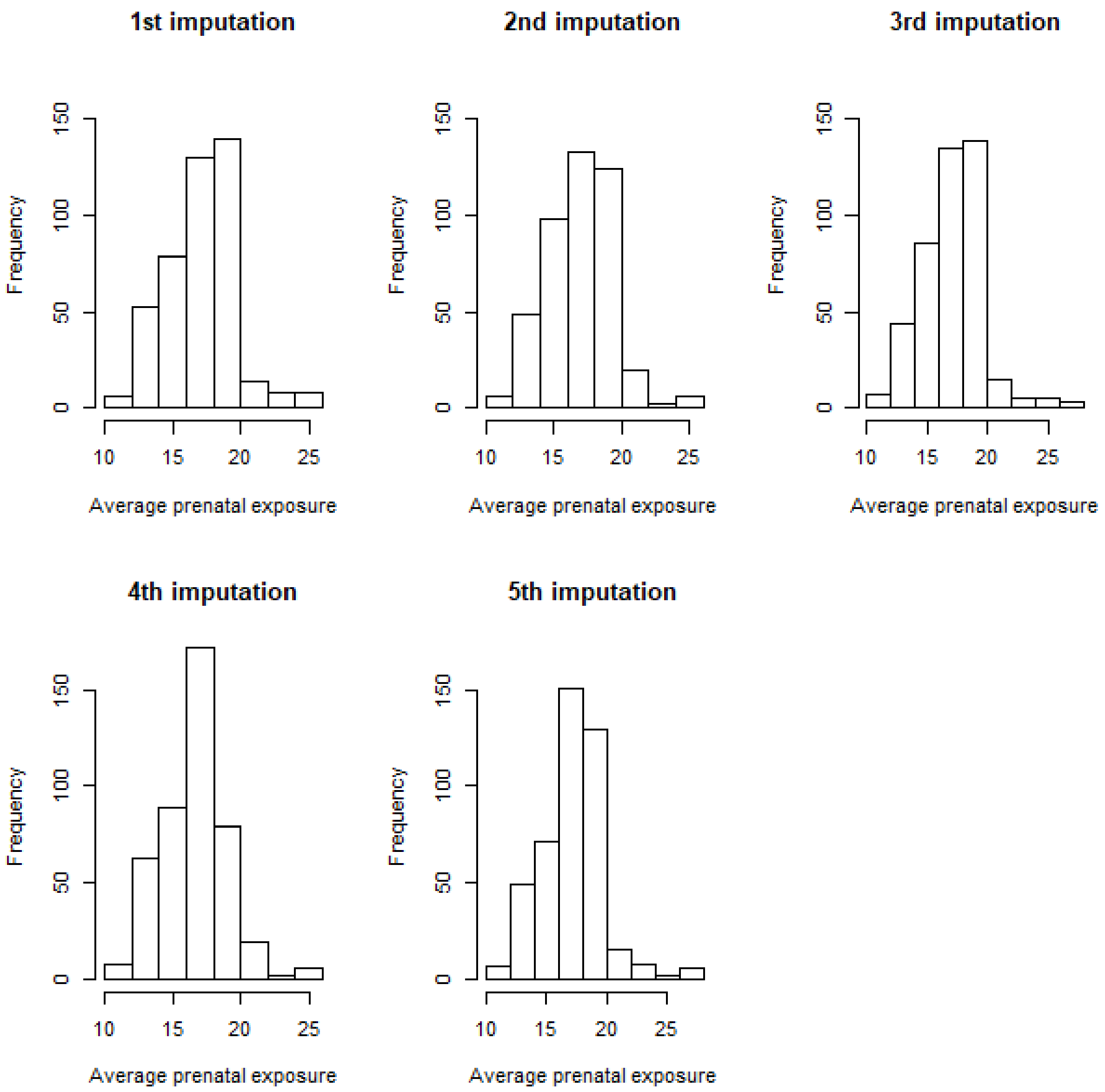

Figure S2. Distribution of the average prenatal exposure to $PM_{2.5}$, by imputed data set.
